# Green method for 17-hydroxyprogesterone extraction and determination using PDMS stir bar sorptive extraction coupled with HPLC: optimization by response surface methodology

**DOI:** 10.1038/s41598-024-66355-9

**Published:** 2024-07-13

**Authors:** Maedeh Noori, Zahra Talebpour

**Affiliations:** 1 Department of Analytical Chemistry, Faculty of Chemistry, Alzahra University, Vanak, Tehran, Iran; 2 Analytical and Bioanalytical Research Centre, Alzahra University, Vanak, Tehran, Iran

**Keywords:** 17-hydroxyprogesterone, Solid phase microextraction, Polydimethylsiloxane, Congenital adrenal hyperplasia, Experimental design, Green chemistry techniques, Biomarkers, Bioanalytical chemistry

## Abstract

Quantifying small amounts of the 17-hydroxyprogesterone in various matrix is crucial for different purposes. In this study, a commercial polydimethylsiloxane stir bar was used to extract hormone from water and urine samples. Analysis was performed by high-performance liquid chromatography using a UV detector. The response surface methodology was used to optimize the desorption and extraction steps, with predicted optimal point relative errors of 1.25% and 6.40%, respectively. The optimized method was validated with a linear range of 1.21–1000.00 for aqueous and 2.43–2000.00 ng mL^–1^ for urine samples. The coefficient of determination was 0.9998 and 0.9967, and the detection limit of the proposed method was obtained to be 0.40 and 0.80 ng mL^–1^ for aqueous and urine samples, respectively. The recovery percentage and relative standard deviation within a day and between three days after the addition of three different concentration levels of the standard to the control sample were 87–103% and 0.4–3.6% for aqueous and 87.5–101% and 0.1–5.2% for urine samples, respectively. The results show that the proposed method can be appropriate and cost-effective for extracting and analyzing this hormone. In addition, using three different tools, the greenness of the proposed method was proven.

## Introduction

17-hydroxyprogesterone (17-OHP) is one of the steroid hormones produced as a mediator in the production of glucocorticoids and sex steroids in the adrenal gland. This hormone is the most important marker for diagnosing and monitoring congenital adrenal hyperplasia (CAH)^1,2^. CAH is an autosomal recessive disease caused by defects of the enzymes involved in the biochemical steps of cortisol production from cholesterol^3^. In patients with CAH (classical and non-classical), the concentration of the 17-OHP hormone in blood serum and urine samples would be higher than the normal level^4,5^. Early diagnosis and timely treatment would prevent the patients from being exposed to excessive androgens, clinical complications after birth, and premature puberty^5,6^. On the other hand, steroid hormones are chemically stable and have high physiological activity at very low concentrations. Steroid hormones are considered an important class of pollutants in aquatic environments, and even their small amounts can cause serious disturbances in the lives of humans and other living creatures that use these waters^7–9^. Moreover, it has been reported in previous studies that 17-OHP is present in runoff and manure^9^. Therefore, a method that can determine small amounts of this hormone in environmental samples and biological fluids would be essential^10^.

Immunoassays are commonly used to measure the levels of adrenal steroids. Due to the non-specific function of antibodies and their reaction with other steroids, the concentration of 17-hydroxyprogesterone is estimated to be higher than its actual level by this method, which causes a misdiagnosis of CAH disease^11–13^. For this reason, more selective methods such as gas chromatography coupled with mass spectrometry (GC–MS)^14^, high-performance liquid chromatography (HPLC)^15^, and liquid chromatography coupled to mass spectrometry (LC-MS/MS)^16^ are considered as more specific techniques for the measurement of steroid hormones because of their incredible power of separation and confirmation of the structures^17^. Techniques such as LC-MS/MS provide suitable detection limits to achieve the mentioned goals. Because it is necessary to perform this analysis in medical diagnostic laboratories on routine tests, the application of common techniques (such as HPLC) coupled with a suitable miniaturized extraction technique for reducing the matrix effect, enrichment of the target compounds, and improving the detection limit of the methods would be of great interest.

In order to merge and miniaturize sample preparation and extraction steps and to reduce the amount of toxic reagents, the novel technique of fiber solid-phase microextraction (SPME) was introduced to eliminate the limitations of the solid phase extraction (SPE) and liquid–liquid extraction (LLE)^18–20^. Several solid-phase microextraction techniques with multiple configurations have been introduced in recent years based on diffusion by stirring or flow^21^. Thin film microextraction (TFME)^22–24^, rotating-disc micro-solid phase extraction^25^, and stir bar sorptive extraction (SBSE)^26–28^ are among the methods based on diffusion by stirring. SBSE was first introduced by Baltussen et al. in 1999 as a novel sample preparation method that allows extraction and concentration in one step^26^. This method has gained much attention in recent years due to its advantages, such as simplicity, high adsorption capacity, robustness, and excellent extraction efficiency. Currently, there are three commercial coatings for SBSE: polydimethylsiloxane (PDMS), polyacrylate (PA), and ethylene glycol-silicone (EG-silicone). The PDMS coating allows the extraction of non-polar species (log K_ow_ > 3) with a very good detection limit, down to sub-nanogram per liter concentrations^29–31^. Consequently, it has been used in analyses of various samples, including environmental matrices, food and biological samples, etc^32^. In the SBSE method, the basis of the work consists of two essential steps: the extraction of analytes from the sample on the sorbent and subsequently, the desorption of the analytes from the sorbent to the desorption solution. The experimental parameters contribute to the extraction efficiency must be adjusted at the best levels to achieve maximum efficiency. Response surface methodology (RSM) is the most appropriate method to simultaneously identify the effective factors in each experiment, find the optimal conditions for the effective factors, and reduce the time and cost spent. According to previous publications, optimization by the RSM is highly effective in optimizing conditions of the SBSE method^33–36^.

Since most of the methods that have been introduced in recent years for the analysis of 17-OHP are based on mass detectors such as LC-MS or LC-MS/MS, and this equipment is not available in all medical diagnostic laboratories, the purpose of the present study was to measure the 17-OHP levels using the SBSE joined to HPLC with an ultraviolet detector (SBSE-HPLC-UV), which has not been previously used to determine this hormone until now. PDMS stir bar was used to extract 17-OHP from water and urine samples. To optimize the desorption and extraction conditions, the RSM was used, and the suitable model was evaluated using the obtained plots and analysis of variance (ANOVA). Eventually, the greenness profile of the proposed method was shown and compared with three different tools, including the analytical eco-scale (AES)^37^, the green analytical procedure index (GAPI)^38^, and the analytical GREEnness (AGREE) metric^39^.

## Experimental

### Chemicals

The Standard of 17-OHP was purchased from Sigma-Aldrich (Steinheim, Germany). Sodium chloride and 1-methyl-3-octylimidazolium tetrafluoroborate ([Omim][BF_4_]) ionic liquid were obtained from RFCL Limited (New Delhi, India) and Kimia Exir (Tehran, Iran), respectively. Perchloric acid (PCA), sulfosalicylic acid (SSA), trichloroacetic acid (TCA), and HPLC-grade methanol (MeOH) were purchased from MERCK (Darmstadt, Germany). Acetic acid and sodium acetate were obtained from RANKEM (New Delhi, India), and HPLC-grade acetonitrile (ACN) was prepared from Fisher Scientific (Loughborough, United Kingdom). HPLC-grade deionized water used in all steps was provided by the Direct-Q system, Millipore (St Quentin, France).

PDMS (Twister^®^, 0.5 mm thickness, 10 mm length, and 24 μL volume) and EG-Silicone (Twister^®^, 0.5 mm thickness, 10 mm length, and 32 μL volume) stir bars were obtained from Gerstel (Muelheim an der Ruhr, Germany). In the first use, the PDMS stir bar was placed in 1 mL of ACN/MeOH solution (80:20, v/v) for 24 h on a magnetic stirrer at 1000 rpm, and the EG-Silicone stir bar was placed in a vial containing 1 mL of ACN for 24 h on a magnetic stirrer at a speed of 1000 rpm.

Between the extractions, the used PDMS stir bars were conditioned in 1.5 mL of ACN and were ultrasonically regenerated for 20 min to remove probably remaining analytes, while the applied EG-Silicone stir bar was introduced into a glass vial containing 0.5 mL of MeOH and was ultrasonically regenerated for 15 min.

### Instrumentation

An HPLC instrument manufactured by Young-Lin Company (Anyang-Si, Korea) with a YL9110 Quaternary pump, 20-µL loop volume, and YL9120 UV detector was used in this study. YL-Clarity software was employed to record chromatograms and integrate the peak areas. The separation was performed on a C_18_ column (5 µm, 4.6 mm i.d. × 150 mm, Phenomenex (Torrance, CA, USA)).

To develop the HPLC-UV method for the analysis of 17-OHP and based on the characteristics of the target compound, three different types of mobile phases, including ACN:H_2_O 53:47 v/v and MeOH:H_2_O 60:40 v/v were examined. Also, the UV wavelength of the detector was set at 240 and 254 nm. Finally, considering the shorter retention time, better peak shape according to the tailing factor, and suitable separation efficiency based on a number of theoretical plates, a mixture of MeOH:H_2_O 60:40 v/v at a flow rate of 1.0 mL min^-1^ was selected as the mobile phase. Also 240 nm was fixed in the UV detector due to the obtained higher peak area (Table S1).

### Preparation of standard and real sample solutions

A 1 mg mL^–1^ stock solution of 17-OHP was prepared in methanol. This solution can be stored at − 20 °C for 6 months. The working standard solutions with lower concentrations were prepared by diluting the stock solution with deionized water, and they can be stored in the refrigerator for a month. At the optimization step, the experiments were carried out with an aqueous solution spiked by 17-OHP at 500 ng mL^–1^ concentration level.

Water real samples were prepared from two sources: tap and well water. Urine samples were collected at 24 h from 6 healthy volunteer people. The sample container was placed in a refrigerator or ice bath during sample collection. To prepare urine samples for extraction, the samples were diluted two times with pure water. The method for urine analyses was carried out in accordance with the principles of the Declaration of Helsinki. Informed consent was obtained from all healthy volunteer people. This study was carried out based on the Master's degree proposal approved by the Alzahra University.

### Comparison of two sorbents in the SBSE method for extraction of 17-OHP

Since the reported value of log p for 17-OHP hormone is 3.17, which is on the border between polar and non-polar properties, PDMS and EG-Silicone sorptive phase were selected to investigate their efficiencies for extraction of 17-OHP during a SBSE method. First, they were prepared to compare the performance of these two coated stir bars as described in Sect. "Chemicals". Then, the procedure was carried out using both under the same conditions. In the extraction step, each stir bar was placed in 30 mL of the 500 ng mL^–1^ 17-OHP standard solution containing 5 g of NaCl for 2 h on a magnetic stirrer at room temperature with a speed of 750 rpm. To desorb the 17-OHP, stir bars were sonicated for 15 min in 250 μL of MeOH/ACN 50:50 v/v solution. All experiments were performed in triplicate.

### Optimization of SBSE procedure

First, the effect of the desorption solvent type, and the addition of a modifier in the desorption solvent were optimized separately by a one-at-a-time approach. Then, the effect of other factors on desorption and extraction efficiencies was evaluated using RSM.

The desorption temperature (20–50 °C) and time (10–30 min) were optimized using full factorial design. Since the suggested solvents for desorption were volatile, the maximum temperature range was selected as 50 °C, and because of the desorption time was not too long, the maximum time range was selected as 30 min. The number of experiments by this design, considering two separate blocks (using two different PDMS stir bars) and three levels, were obtained to be 18 experiments. In this design, two responses were considered: the peak area after the first desorption and the peak area after re-desorption without performing the extraction step again (memory effect). The obtained chromatograms were recorded after each experiment, and the desorption step was optimized according to the results. To optimize the extraction step, the face-centered central composite design (FCCD) was used at three levels with ten repetitions at the central point. The advantage of using the CCD method is that it shows the effects of main factors and interactions well and provides useful information about them. In addition, the CCD method also reduces the number of experiments required for optimization related to the three levels full factorial design. Among the different types of CCD methods, FCCD has the advantage that it does not place the experimental design points outside the selected operating range^40^. In this step, pH (6–8), NaCl content (0–20 w/v%), sample volume (10–30 mL), extraction temperature (25–50 °C), and extraction time (30–150 min) were selected as the factors affecting the extraction efficiency, and peak area of 17-OHP after the first desorption step at the optimal conditions was recorded as the response. All the design of the experiments and their evaluation based on the ANOVA results were performed using the Statgraphics Centurion XV software (version 18). In addition, Pareto, relative residual, and response surface charts were drawn, and the effect of each factor was investigated.

According to the evaluated results, the optimized SBSE procedure was explained as follows: the stir bar was placed in 10 mL of the sample solution with 20% NaCl for 80 min, at a temperature of 25 °C and 750 rpm stirring speed, without adjusting the pH. Then, the stir bar was removed from the solution, dried with a clean, lint-free tissue, placed in 250 μL of MeOH:ACN 50:50 v/v, and sonicated for 30 min at 50 °C. Finally, 20 μL of desorbed solution was injected into HPLC.

### Method validation

After optimization of the extraction and desorption conditions to determine 17-OHP, the proposed method was validated. Validation of the method in this project was done using the international published reference guidelines^41,42^. In order to ensure the selectivity of the method, the blank solutions of water and urine were prepared from 6 different sources and were subsequently injected into the HPLC after the extraction. The calibration curve of water and urine samples were drawn and evaluated at eight concentration levels of 10 to 1000 and 20 to 2000 ng mL^–1^, respectively. Since the urine samples were diluted with water, the concentration of the original urine sample was considered to be twice the concentration of the corresponding aqueous sample to draw the calibration curve. To calculate the LOD and LOQ values, six extractions were carried out from the blank sample (pure water for the water samples and diluted urine for the urine samples) under the obtained optimal conditions. After calculating the standard deviation of the blank sample (σb) and using the slope of the calibration curve (S), the LOD and LOQ values were obtained according to the following relationships:

1 $$LOD = \text{=} \frac{\text{3.3}{\sigma }_{b}}{\text{S}}.$$

2 $$LOQ=\frac{\text{10 }{\sigma }_{b}}{\text{S}}.$$

To calculate the accuracy and precision of the method, water samples at three concentrations of 30, 400, and 800 ng mL^–1^, as well as the urine samples at three concentrations of 60, 800, and 1600 ng mL^–1^, were extracted and analyzed in triplicate under optimum conditions for each of the concentrations during one day and three consecutive days. The intra- and inter-day accuracy values of the proposed method were evaluated by calculating the recovery percentage, whereas precision was reported by the relative standard deviation (RSD%). In addition, according to the concentration factor (CF) and enrichment factor (EF), the extraction efficiency (EE) was obtained using the following equations:

3 $$\text{CF = }\frac{{\text{V}}_{1}}{{\text{V}}_{2}} ,$$

4 $$EF = \frac{{\text{C}}_{2}}{{\text{C}}_{1}},$$

5 $$\text{EE} = \frac{\text{EF}}{{\text{CF}}},$$

C1 and C2 represent the initial and final concentrations of 17-OHP in the sample and desorption solution, and V1 and V2 stand for the volume of the sample and desorption solution, respectively.

### Evaluation of the greenness of the PDMS-SBSE-HPLC–UV method

To evaluate the degree of the greenness of the method, three tools, analytical eco-scale (AES)^37^, green analytical procedure index (GAPI)^38^, and Analytical GREEnness metric (AGREE)^39^ were used. In the eco-scale technique, the parameters affecting the method's greenness are divided into reagents and instruments. From the sum of the penalty points for each factor, the final penalty point value is obtained, then this value is subtracted from 100, and the obtained number would be an indicator of the degree of greenness of the method. If the total penalty point is > 75, the method is "excellent" in terms of greenness; the value > 50 indicates that the method is acceptable, while the value of 50 shows an insufficiently green method. In the GAPI metric, 15 factors are considered to measure the degree of greenness of the method, and each of these factors are divided into three categories, including green, yellow, and red, according to their values. Using this tool, the obtained results were shown in the form of a pictogram, and the presence of a circle in the central pentagon indicated the quantitativeness of the method. AGREE software^39^ was also used as the last tool. In this technique, each parameter affecting the degree of greenness has a value between zero and one. The total average value was obtained, and the closer it is to one, the higher the level of greenness of the method.

## Results and discussion

### Investigating the efficiency of sorbents in the SBSE method for extraction of 17-OHP

**Figure 1:**
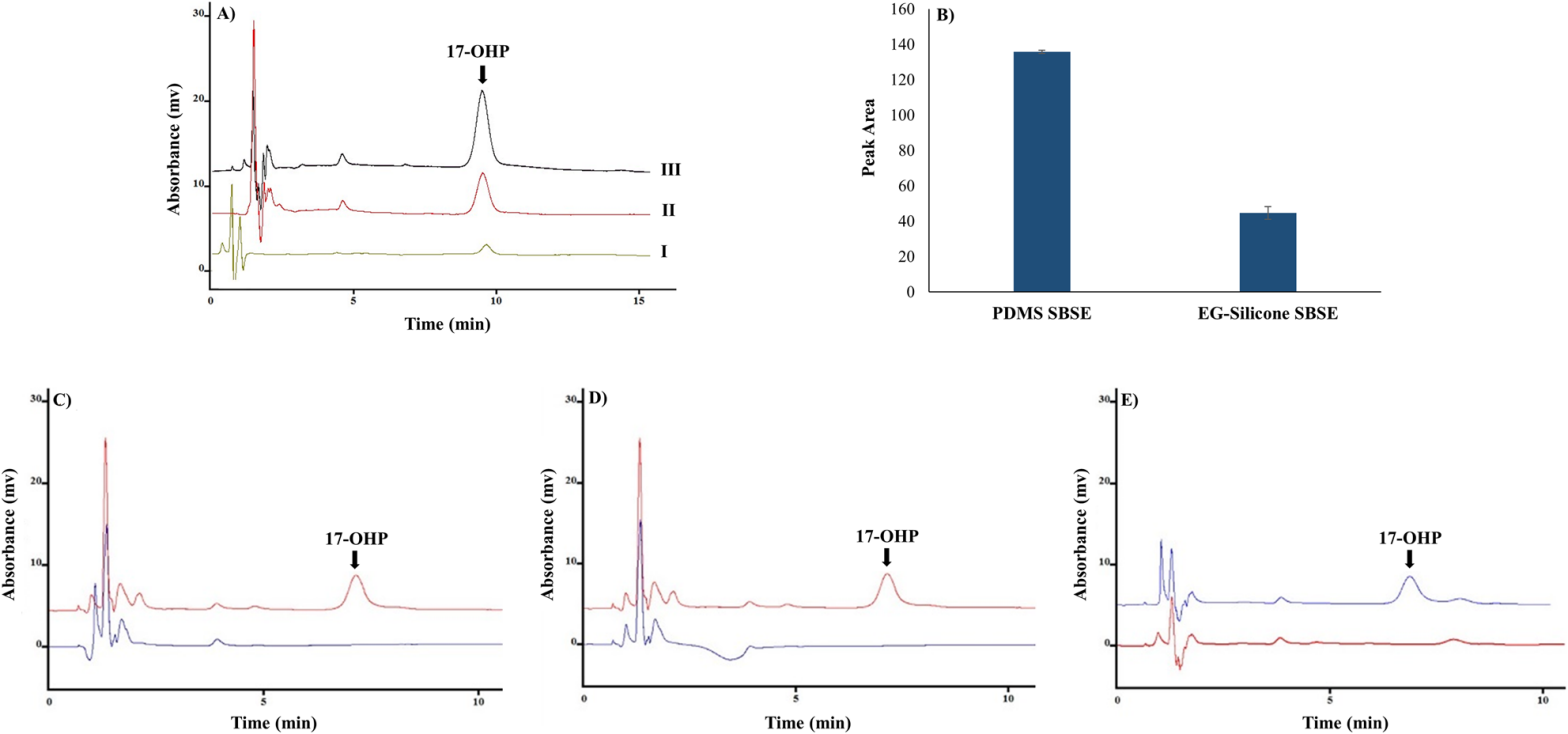
(**A**) Comparison of the chromatograms of 17-OHP standard solution at the concentration level of 500 ng mL^–1^ without extraction (I), extraction by EG-Silicone stir bar (II), and extraction by PDMS stir bar (III). (**B**) Comparison of the extraction of 17-OHP (30 mL of 500 ng mL^–1^ solution) using PDMS and EG-Silicone stir bars under constant extraction conditions with three replications. Chromatograms obtained from the analysis of 17-OHP hormone in real samples (**C**) tap water, (**D**) well water, and (**E**) urine before (bottom) and after (top) addition of 500 ng mL^–1^ of 17-OHP to sample by PDMS-SBSE-HPLC-UV method:

The effect of sorbent type (PDMS and EG-Silicons) in the SBSE method to extract 17-OHP was investigated as described in Sect. "Comparison of two sorbents in the SBSE method for extraction of 17-OHP". The desorption solutions were analyzed using HPLC instrument, and the obtained chromatograms and the related peak areas of 17-OHP using PDMS and EG-Silicone sorptive phase are depicted in Fig. 1. As is apparent in Fig. 1A, the extractions using PDMS and EG-Silicone stir bars displayed increased peak areas of 17-OHP compared to the result obtained by direct injection of the same concentration. Moreover, according to the peak area of 17-OHP (Fig. 1B), the PDMS stir bar performed much better than the EG-Silicone to extract the 17-OHP hormone. Based on the chemical structures of 17-OHP and the two sorbents used, it seems that the hydrophobic interactions between 17-OHP and the PDMS were more effective than the dipole–dipole interaction of hydroxyl groups in EG-Silicone with 17-OHP. Thus, PDMS was selected as the suitable sorptive phase for the rest of the experiments. Also, the performance of the stir bars in real samples of tap water, well water and urine were investigated, which is shown in Fig. 1C–E. As can be seen after spiking of 500 ng mL^–1^ 17-OHP to each real sample, its peak was appeared in the chromatogram without interference.

### Optimization of the SBSE procedure

#### Optimization of desorption conditions

**Figure 2:**
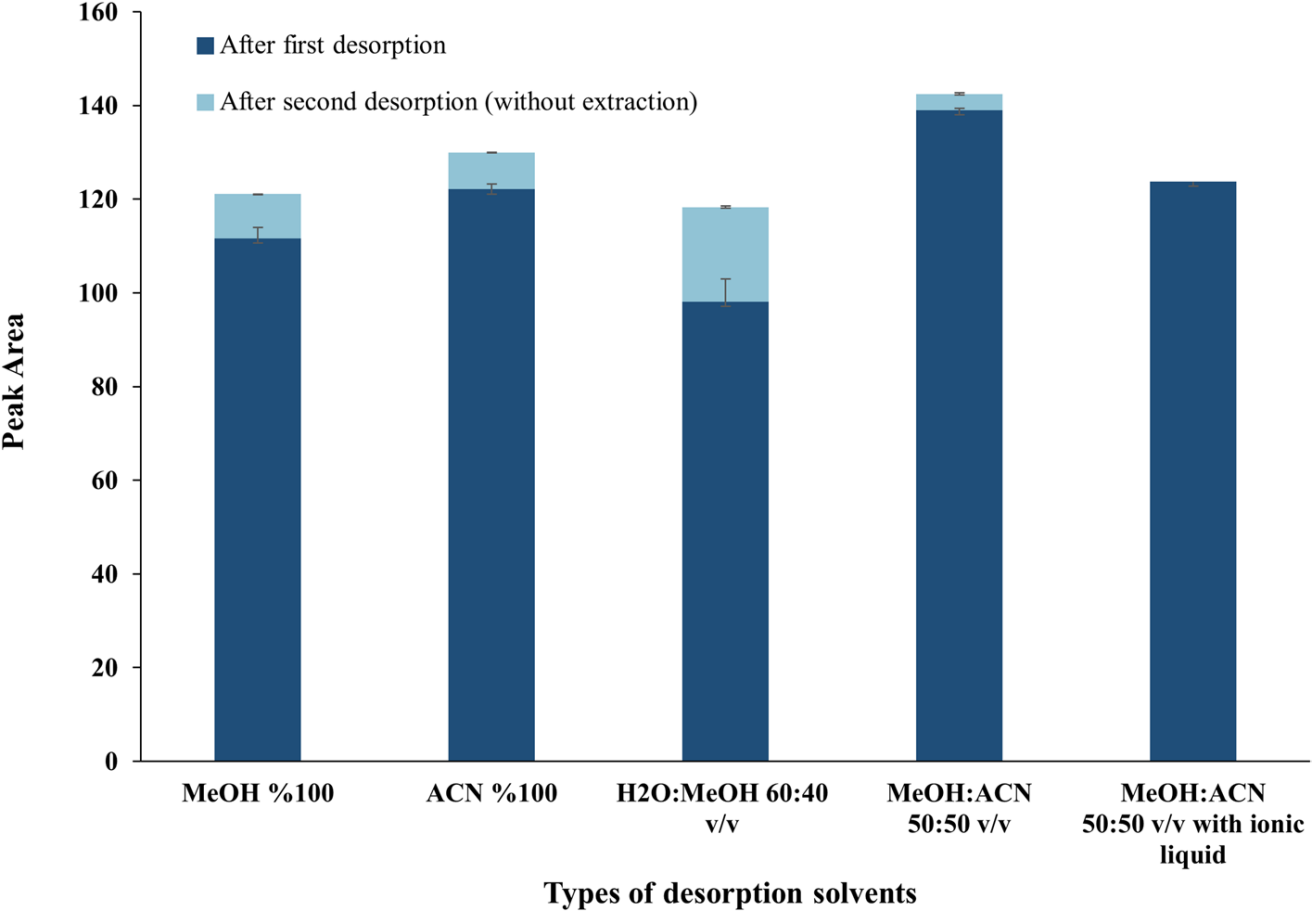
Comparison of different desorption solvents to optimize desorption solvent type and proportion (v/v) for extracting 30 mL of 500 ng mL^–1^ solution of 17-OHP under constant conditions.

As explained in Sect. "Optimization of SBSE procedure", one factor at a time for optimization of desorption solvent and full factorial for optimization of other factors were used to find the best desorption conditions. The type of desorption solvent was optimized using four different solvents (MeOH, ACN, H_2_O:MeOH 60:40 v/v, and MeOH:ACN 50:50 v/v), and the effect of a polar ionic liquid in the desorption phase was also investigated. To this end, 30 mL of the standard sample of 17-OHP with a concentration of 500 ng mL^-1^ was first prepared, and 5 g of NaCl was added to it. Then, the solution was extracted for 2 h using a PDMS stir bar at a speed of 750 rpm at room temperature. After the extraction step, the stir bars were placed in 250 μL of 5 different solutions in an ultrasonic bath for 15 min. As shown in Fig. 2, the best solvent for desorption was MeOH/ACN 50:50 v/v. Due to the combination of two solvents (ACN and MeOH), dipole–dipole and hydrogen bonding interactions with the analyte can be established. Thus, it has acted more strongly for the desorption of the analyte compared to the use of each of these solvents alone. On the other hand, the solubility of the analyte in methanol (organic phase) is higher than that in water; hence, the desorption efficiency in MeOH:H_2_O 40:60 v/v would be lower than other solvents. According to previous experiments^43^, the [Omim][BF_4_] ionic liquid was also used as a modifier in the optimized desorption solvent; however, as shown in Fig. 2, the addition of the ionic liquid did not have a positive effect on the extraction efficiency.

**Figure 3:**
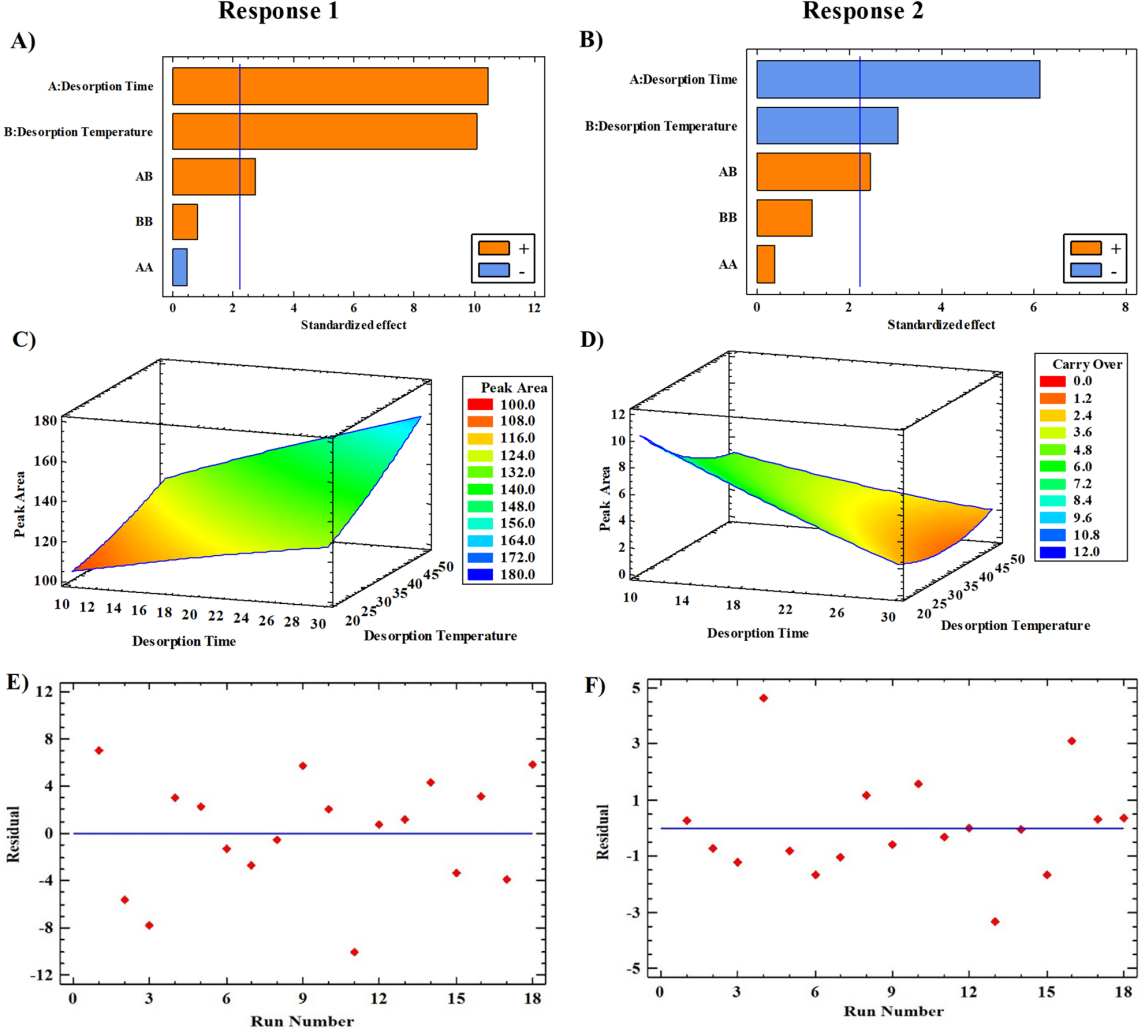
Plots related to optimizing the desorption step and memory effect using a three-level full factorial design. The left side illustrates the optimization graphs of response one, and the right side depicts the optimization graphs of response two; (**A**) and (**B**) Pareto, (**C**) and (**D**) response surface, and (**E**) and (**F**) relative residual plots according to experiment number.

A 3-level full factorial design was used considering two blocks (two stir bars) to optimize the desorption time and temperature. The design matrix and obtained responses were represented in Table S2. The extraction conditions were considered constant during the optimization steps. In optimizing the desorption step, the values of the peak areas after the first desorption were considered as response one, and the peak areas obtained after the second desorption (without performing the extraction step again) for investigating the memory effect were considered as response 2. The changes of peak area according to the conditions of each experiment were analyzed. The main and binary interaction effects of factors for both responses were individually calculated, and their significance was investigated using ANOVA, which is reported completely in the supplementary material (Tables S3 and S4) for the two responses. According to the *p*-value < 0.05 and Pareto charts (Fig. 3A and B), desorption time (A), desorption temperature (B), and their interactions (AB) had significant effects on both responses. Moreover, the quadratic variables (AA and BB) did not significantly affect responses 1 and 2. It rejects the existence of curvature in the relationship between these factors and both responses. Moreover, the non-significance block effect indicated that the use of different stir bars did not significantly affect the extraction efficiency.

The value of R^2^ also indicated that the model for response 1 describes 95.77% of the results and was very close to the R^2^_adj_ value (93.23). As is apparent from the response surface plots (Fig. 3C and D), and the amount of desorption increased by increasing the desorption time and temperature. At the same time, the value of memory effect decreased. In fact, by raising the temperature, diffusion into the sample increases, and the analyte would be transferred faster from the sorbent to the solution. The relative residual plots (Fig. 3E and F) for both responses also displayed a random distribution of data and the suitability of the models designed to optimize the desorption step. According to evaluated models, the optimal values for desorption time and temperature to maximize the peak area after the first desorption and to minimize the peak area after the second desorption were obtained to be 30 min and 50 °C, respectively. The experimental values obtained under this condition were very close to the predicted values from each response and exhibited a very small error percentage (1.25 and 7.02%), which indicates the validity of the fitted model used to optimize the desorption step for both responses (Table 1).

**Table 1:** The optimum conditions for extraction of 17-OHP from aqueous media using the SBSE method.

Response: peak area of 17-OHP	The optimal levels (in the desorption step)					Predicted peak area	Obtained peak area	% relative error
	Desorption time (min)			Desorption temperature (°C)				
After the first desorption	30			50		166.11	168.19	1.25
After the second desorption	30			50		1.01	1.081	7.02

	The optimal levels (in the extraction step)							
	pH	NaCl (%w/v)	Sample volume (mL)	Extraction temperature (°C)	Extraction time (min)			
Without limiting time	6.00	20.00	10.01	49.96	121.71	198.56	185.85	6.40
Limiting time to 90 min	6.94	19.98	10.10	25	80.85	162.36	135.66	16.44

#### Optimization of extraction condition

To optimize the extraction step, FCCD was used as described in Sect. "Comparison of two sorbents in the SBSE method for extraction of 17-OHP", and the corresponding design matrix is represented in Table S5. Since different blocks did not significantly affect the desorption step's optimization, the block effect was not considered in this design. The ANOVA results of the main and interaction factors are reported in Table S6. The results revealed that the main factors, including NaCl content (B), sample volume (C), extraction temperature (D), and extraction time (E), as well as the interaction factors, including AB, AC, BD, BE, CE, and DE, and quadratic parameters such as AA, BB, CC, DD, and EE, have a significant effect (*p-value* < 0.05) on the extraction efficiency at the confidence level of 95%.

To investigate the effect of each of the factors, Pareto charts were also drawn (Fig. S1). Based on these results, NaCl content, temperature, and time positively affect the extraction efficiency, whereas the sample volume negatively affects it. The most significant effect was NaCl content because increasing the ionic species in the sample reduces the amount of water molecules to dissolve the analyte, and the conditions for extraction of the analytes to the sorbent would become more favorable^44^. Extraction temperature has two opposing effects on SBSE: the equilibrium is reached faster at higher temperatures; however, at the same time, the solubility of the analytes in water would increase (K_o/w_ decreases), which leads to a decrease in extraction efficiency. As shown in the results, increasing the extraction temperature in this study positively affected extraction efficiency. Based on the results obtained in this design, the effect of changing the sample volume on the extraction efficiency was negative, because by reducing the sample volume, the extraction kinetics were improved and the extraction reached the equilibrium faster^45^. It is also obvious from the Pareto chart that although the pH value did not significantly affect the extraction efficiency, its interactions with NaCl content and sample volume were significant.

Some of the response surface plots resulting from the central composite design to optimize the extraction step are depicted in Fig. S2A–E. In each plot, two factors were considered supposed variable factors, and the remaining factors were considered fixed at the middle level. The values of R^2^ and R^2^_adj_ of the predicted model were 99.84% and 99.58%, respectively, which were very close to each other and indicated that this model described more than 99% of the results well and is a suitable model for optimizing the extraction conditions. Moreover, the relative residual plot of the proposed model is illustrated in Fig. S2F. Due to the random distribution of the residues, it was confirmed that the designed model is suitable for optimizing the factors.

The optimal value for each factor was obtained from the predicted model (reported in the Supplementary file), and the results are listed in Table 1. The best extraction time was predicted to be approximately two hours (close to the equilibrium conditions), and the relative error percentage of the obtained results was appropriate (6.40%). Since 17-OHP is on the border between polar and non-polar properties, a longer extraction time would be required to reach equilibrium than non-polar compounds. Under this situation, kinetically-controlled extraction (i.e. extraction at constant times less than the time needed to reach the equilibrium) can be applied^46^. To this end, the extraction time was kept constant at 90 min, and the optimum levels for other factors were found according to the proposed model (Table 1).

### Method validation

Considering that the aim of the present study was the analysis of 17-OHP in aqueous media and urine, the selectivity of the method was investigated using real samples. The obtained chromatograms related to the analysis of six different real samples of water and urine before and after the spike of 500 ng mL^–1^ 17-OHP to the blank samples of water and urine are depicted in Fig. S3. Since there were no interfering peaks in water and urine samples at the retention time of 17-OHP (7 min), the method exhibited good selectivity.

Under the optimum conditions, the calibration curves were separately obtained for blank water and urine samples at the concentration levels mentioned in Sect. “Optimization of SBSE procedure”. Using the least-squares method, equations of the lines and the correlation coefficients were obtained. Validation parameters to determine 17-OHP in water and urine are reported in Table 2. The values of R^2^ for calibration curves prepared in water and urine were calculated to be 0.9998 and 0.9967, respectively. The ANOVA table (Table S7) indicated that the F value was greater than the F critical at a 95% confidence level and the regressions were significant for both calibration curves. In addition, the lack of fit (LOF) F values for both calibration curves (4.200 and 3.870 for water and urine, respectively) were smaller than F critical at a 99% confidence level (4.695 and 4.202), there was no significant LOF and the models were suitable for the calibration curves (Table S7). The linearity range of the method was observed from 1.21 to 1000.00 and 2.43 to 2000.00 ng mL^–1^ for water and urine samples, respectively. LOD and LOQ values were calculated using Eqs. (1) and (2), which were obtained to be 0.40 and 1.21 ng mL^–1^ for water samples and 0.80 and 2.43 ng mL^–1^ for urine samples, respectively.

**Table 2:** Analytical performance for the determination of 17-OHP in water and urine samples.

Sample	Line equation	Linear range (ng/mL)	Coefficient of determination (R^2^)	LOD^a^ (ng/mL)	Added amount (ng/mL)	Intra-day (n = 3)		Inter-day (n = 3)	
						RSD^b^ (%)	Recovery (%) ± SD^c^	RSD (%)	Recovery (%) ± SD
Water	Y = 0.24 x + 4.08	1.2–1000.0	0.999	0.4	30	3.6	91.0 ± 3.6	1.4	87.0 ± 3.6
					400	0.9	102.9 ± 0.9	1.0	101.8 ± 0.9
					800	0.4	99.6 ± 0.4	0.6	99.5 ± 0.4
Urine	Y = 0.08 x + 6.48	2.4–2000.0	0.997	0.8	60	2.3	87.5 ± 2.3	5.2	93.6 ± 5.2
					800	1.6	99.0 ± 1.6	2.5	100.8 ± 2.5
					1600	0.1	99.5 ± 0.1	0.6	99.8 ± 0.6

^a^Limit of detection, ^b^Relative standard deviation, ^c^Standard deviation.

The proposed method's intra- and inter-day accuracy and precision in water and urine media were obtained based on the mentioned procedure in Sect. "Optimization of SBSE procedure". The results (reported in Table 2) demonstrated excellent recovery values in the ranges of 87–103% and 87.5–101% for water and urine samples, respectively. According to the obtained results, the RSD% values for the two media were within the ranges of 0.4–3.6% and 0.1–5.2%, respectively; indicating good precision of the proposed method for the analysis of 17-OHP in water and urine. CF, EF, and EE% were also calculated according to the Eqs. (3)–(5) at 100 ng mL^-1^ concentration level of 17-OHP, which were obtained to be 40, 13, and 32.5, respectively.

### Real sample

To demonstrate the efficiency of the PDMS stir bar for extraction of 17-OHP from real samples, tap water, well water, and urine samples of a healthy person were tested. A standard solution of 17-OHP with a concentration of 500 ng mL^–1^ was spiked to each of the water and urine samples, and the extractions were performed under optimal conditions for real and spiked samples. The obtained chromatograms (before and after the spike of standard solution to each real sample) are depicted in Fig. 1C–E. The recovery and RSD% were also calculated for real samples, reported in Table S8. As can be seen, for two samples of tap water and well water as well as a urine sample, the recovery values were in the range of 98–105%, and the RSD% was found to be ≤ 1, which confirmed the effectiveness of the proposed method for extraction and analysis of this hormone from these media.

### Evaluation of the greenness of the PDMS-SBSE-HPLC–UV method

As mentioned in Sect. "Evaluation of the greenness of the PDMS-SBSE-HPLC–UV method", in this study, three tools, AES, GAPI and AGREE, were used to check the greenness of the proposed method. The principles of calculating the amount of greenness with each of the methods were briefly explained in Sect. "Evaluation of the greenness of the PDMS-SBSE-HPLC–UV method". The results obtained in Table 3 showed that since the amount of penalty points obtained using the AES method was equal to 86, the method proposed in this article was placed in the "excellent" category in terms of the amount of greenness. In the investigation using GAPI method, as shown in Table 3, since most of the parts of the pentagons in the obtained pictogram were green and yellow, it can be concluded that the proposed method in this study was green. AGREE was used as the last method to check the degree of greenness. The calculations of this method were done using AGREE software and the result is reported in Table 3. Since the value obtained using this method was closer to one and was higher than 0.5, the greenness of the method was also confirmed by the AGREE method.

**Table 3:** Evaluation of the greenness of the PDMS-SBSE-HPLC-UV method using eco-scale, GAPI and AGREE tools.

Eco-scale		GAPI	AGREE
Reagents	PPs^a^		
Water	0		
Methanol	6		
Acetonitrile	4		
NaCl	0		
Instruments			
Energy	1		
Occupational hazard	0		
Waste	3		
Total PP	14		
Eco-scale	86		

^a^Penalty points.

### Comparison of the introduced method with previous methods

The method proposed in this study was compared to other methods used to analyze 17-OHP (Table 4)^47–52^. Since the HPLC–UV technique was used in the present investigation, higher detection limit values in comparison with those obtained by LC–MS/MS might be expected; however, the obtained detection limit for the proposed method exhibited appropriate value for diagnosis of CAH disease via the analysis of 17-OHP in urine^4,14,52,53^. Moreover, the RSD value of the method was less than 5.2%, and the stir bars demonstrated repeatable results for multiple extractions up to 18 independent extractions when the RSD value was less than 6.4% (Fig. S4). The method proposed in this study utilizes the HPLC technique and a commercial sorbent for extraction. This method is more cost-effective compared to other methods such as LC–MS/MS and UPLC-MS/MS which has been used to determine this hormone in other reported articles (Table 4). Recently, Manousi et al. have clearly shown that methods using HPLC–UV techniques are considered simple and readily available in most laboratories. However, methods using LC–MS/MS or UPLC-MS/MS are categorized as not being normally available in most laboratories^54^. On the other hand, previous studies have not evaluated the degree of greenness of their methods. According to the parameters that are important in calculating the greenness of a method with the help of tools such as AGREE, Eco-Scale, and GAPI, the previous methods consume a large amount of solvent due to the use of extraction techniques such as SPE and LLE.

**Table 4:** Comparison of reported methods for determination of 17-OHP.

Sample	Extraction method	Analysis method	LOD^a^ (ng/mL)	LDR^b^ (ng/mL)	References
Serum	SPE^c^	LC-MS/MS	0.03	0.05–26.44	^45^
Serum	LLE^d^	UPLC-MS/MS	0.1	0.5–300	^46^
Dried blood spots	LSE^e^	LC-MS/MS	0.1	0.1–67.5	^47^
Dried blood spots	LLE	LC-MS/MS	5	ND^f^	^48^
Dried blood spots	LSE	LC-MS/MS	1	1–500	^49^
Urine	SPE	LC-MS/MS	0.25	0.5–250	^50^
Urine	SBSE^g^	HPLC-UV	0.8	2.43–2000	This study

^a^Limit of detection, ^b^Linear dynamic range, ^c^Solid phase extraction, ^d^Liquid-liquid extraction, ^e^Liquid solid extraction, ^f^No data, ^g^Stir bar sorptive extraction.

### Conclusion

In this research, a commercial PDMS stir bar joint to HPLC–UV technique was used to determine 17-OHP in water and urine samples. The effective factors in the desorption stage were optimized using a full factorial design and one-factor-at-a-time method. In contrast, the influential parameters during the extraction step were optimized using a central composite design. The method validation results for water and urine samples showed high good recovery and acceptable detection limit (0.40 and 0.80 ng mL^–1^ for aqueous and urine samples, respectively). The coefficient of determination of the introduced method was good (0.9998 and 0.9967 for aqueous and urine samples, respectively). Since radioimmunoassay methods suffer from poor selectivity, according to the obtained results, this method can be considered as good alternative to immunoassay methods for analysis of the selected hormones. On the other hand, due to the use of commercial stir bar and HPLC instrument, the developed method can be a routine and less expensive method compared with LC–MS or LC-MSMS techniques for analyzing this hormone. In addition, the greenness of the proposed method in this study has been proven by using different methods of calculating greenness. Moreover, it can be used to analyze this hormone in other biological fluids, such as blood serum. It is suggested to investigate the use of the proposed method in other environments such as blood serum and plasma in future studies. Also, the use of this method in the medical diagnosis laboratory and checking its effectiveness is one of the things that can be considered.

### Supplementary Information

Supplementary Information.

## Data Availability

Data is provided within the manuscript or supplementary information files.
