# Supplementary material for: Green method for 17-hydroxyprogesterone extraction and determination using PDMS stir bar sorptive extraction coupled with HPLC: optimization by response surface methodology
Source: Sci Rep. 2024 Jul 13;14:16192. doi: 10.1038/s41598-024-66355-9 (PMC11246442; doi:10.1038/s41598-024-66355-9)
Supplement: Supplementary file 1 — Supplementary Information. [file 41598_2024_66355_MOESM1_ESM.docx]

**Green method for 17-hydroxyprogesterone extraction and determination using PDMS stir bar sorptive extraction coupled with HPLC: Optimization by response surface methodology** Maedeh Noori^1^, Zahra Talebpour^1, 2, *^

^1^ Department of Analytical Chemistry, Faculty of Chemistry, Alzahra University, Vanak, Tehran, Iran

^2^ Analytical and Bioanalytical Research Centre, Alzahra University, Vanak, Tehran, Iran

^*^Corresponding author; E-mail addresses: [ztalebpour@alzahra.ac.ir](mailto:ztalebpour@alzahra.ac.ir); [ztalebpour@yahoo.com](mailto:ztalebpour@yahoo.com)

| **Table S1.** Effects of the analytical parameters in peak area, retention time (Rt), tailing factor (T) and theoretical plates number (N) of the chromatographic method for 17-OHP quantitation. | | | | |
| --- | --- | --- | --- | --- |
| **Effect** | **Peak area (%)** | **Rt^a^ (min)** | **Tailing factor** | **N^b^** |
| **Mobile phase composition**  **A:** MeOH:H_2_O (60:40 v/v)  **B:** ACN:H_2_O (53:47 v/v) | 50.86 $-$ 48.19 $=$ 2.67 | 7.72 $-$ 8.05 $=$ - 0.33 | 1.08 $-$ 1.33 $=$ -0.25 | 8756 $-$ 14187 $=$ -5431 |
| **UV wavelength**  **A:** MeOH:H_2_O (60:40 v/v), 240 nm  **B:** MeOH:H_2_O (60:40 v/v), 254 nm | 50.86 $-$ 19.53 $=$ 31.33 | 7.72 $-$ 7.71 $=$ 0.01 | 1.08 $-$ 1.00 $=$ 0.08 | 8756 $-$ 9288 $=$ -532 |
| **^1^** Retention time  **^b^** Number of theoretical plate | | | | |

| **Table S2.** The design matrix of a 3-level full factorial design for optimization of desorption step and the obtained peak areas of 17-OHP. | | | | | |
| --- | --- | --- | --- | --- | --- |
| **Run order** | **Blocks** | **Desorption Time (min)** | **Desorption Temperature (°C)** | **Response 1:**  **Peak area after the first desorption** | **Response 2:**  **Peak area after the second desorption** |
| 1 | 1 | 10 | 50 | 114.89 | 3.06 |
| 2 | 1 | 10 | 20 | 102.92 | 10.80 |
| 3 | 1 | 10 | 35 | 114.07 | 6.53 |
| 4 | 1 | 20 | 20 | 114.32 | 4.74 |
| 5 | 1 | 20 | 50 | 141.67 | 2.67 |
| 6 | 1 | 30 | 35 | 131.68 | 0.93 |
| 7 | 1 | 20 | 35 | 128.31 | 9.85 |
| 8 | 1 | 30 | 50 | 163.27 | 0.67 |
| 9 | 1 | 30 | 20 | 130.01 | 1.80 |
| 10 | 2 | 30 | 20 | 128.58 | 3.02 |
| 11 | 2 | 20 | 20 | 115.94 | 4.68 |
| 12 | 2 | 10 | 50 | 127.01 | 4.18 |
| 13 | 2 | 10 | 20 | 103.12 | 9.89 |
| 14 | 2 | 20 | 50 | 150.98 | 6.71 |
| 15 | 2 | 30 | 35 | 130.85 | 1.25 |
| 16 | 2 | 20 | 35 | 130.10 | 1.82 |
| 17 | 2 | 30 | 50 | 169.00 | 1.35 |
| 18 | 2 | 10 | 35 | 117.76 | 7.40 |

| **Table S3.**  The ANOVA table of a 3-level full factorial design for optimization of desorption step in an SBSE technique according to response 1 (peak area of 17-OHP after the first desorption). | | | | | | | |
| --- | --- | --- | --- | --- | --- | --- | --- |
| **Factors** | **Sum of square** | **Degree of freedom** | **Mean of square** | ***p***  **value** | **F**  **value** | **Levels of factors** | |
|  |  |  |  |  |  | **Low** | **High** |
| **Desorption Time (min): A** | 2637.38 | 1 | 2637.38 | **0.000** | 109.11 | 10 | 30 |
| **Desorption Temperature (°C): B** | 2436.87 | 1 | 2436.87 | **0.000** | 101.93 | 20 | 50 |
| **AA** | 5.52 | 1 | 5.52 | 0.6429 | 0.23 |  | |
| **AB** | 178.83 | 1 | 178.83 | **0.0216** | 7.40 |  | |
| **BB** | 16.11 | 1 | 16.11 | 0.4333 | 0.67 |  | |
| **Blocks** | 114.69 | 1 | 114.69 | 0.0544 | 4.74 |  | |
| **R^2^** | 95.77 |  |  |  |  |  | |
| **R^2^_adj_** | 93.23 |  |  |  |  | | |

| **Table S4.**  The ANOVA table of a 3-level full factorial design for optimization of desorption step in an SBSE technique according to response 2 (peak area of 17-OHP after the second desorption). | | | | | | | |
| --- | --- | --- | --- | --- | --- | --- | --- |
| **Factors** | **Sum of square** | **Degree of freedom** | **Mean of square** | ***p***  **value** | **F value** | **Levels of factors** | |
|  |  |  |  |  |  | **Low** | **High** |
| **Time (min): A** | 89.71 | 1 | 89.71 | **0.001** | 37.59 | 10 | 30 |
| **Temperature (°C): B** | 22.12 | 1 | 22.12 | **0.012** | 9.27 | 20 | 50 |
| **AA** | 0.34 | 1 | 0.34 | 0.712 | 0.14 |  | |
| **AB** | 14.18 | 1 | 14.18 | **0.035** | 5.94 |  | |
| **BB** | 3.34 | 1 | 3.34 | 0.264 | 1.40 |  | |
| **Blocks** | 1.95 | 1 | 1.95 | 0.387 | 0.82 |  | |
| **R^2^** | 84.58 |  |  |  |  |  | |
| **R^2^_adj_** | 75.32 |  |  |  |  |  | |

| **Table S5.** The design matrix of a face-centered central composite design (FCCD) for optimization of extraction step and the obtained peak areas of 17-OHP. | | | | | | |
| --- | --- | --- | --- | --- | --- | --- |
| **Run order** | **pH** | **Ionic Strength (%w/v)** | **Sample Volume (mL)** | **Extraction Temperature (°C)** | **Extraction Time (min)** | **Peak Areas** |
| 1 | 6 | 10 | 20 | 50 | 90 | 102.10 |
| 2 | 6 | 20 | 20 | 37.5 | 90 | 157.94 |
| 3 | 4 | 0 | 30 | 25 | 30 | 27.35 |
| 4 | 8 | 20 | 30 | 50 | 150 | 182.20 |
| 5 | 4 | 20 | 10 | 25 | 30 | 63.47 |
| 6 | 8 | 20 | 10 | 25 | 150 | 172.05 |
| 7 | 4 | 20 | 10 | 50 | 150 | 186.54 |
| 8 | 6 | 10 | 20 | 37.5 | 90 | 83.66 |
| 9 | 6 | 0 | 20 | 37.5 | 90 | 52.01 |
| 10 | 8 | 0 | 10 | 50 | 150 | 51.56 |
| 11 | 8 | 20 | 10 | 50 | 30 | 74.72 |
| 12 | 4 | 0 | 10 | 25 | 150 | 57.18 |
| 13 | 6 | 10 | 10 | 37.5 | 90 | 91.18 |
| 14 | 4 | 20 | 30 | 25 | 150 | 146.06 |
| 15 | 6 | 10 | 20 | 37.5 | 90 | 83.26 |
| 16 | 6 | 10 | 20 | 25 | 90 | 110.24 |
| 17 | 4 | 0 | 30 | 50 | 150 | 41.60 |
| 18 | 6 | 10 | 20 | 37.5 | 90 | 87.46 |
| 19 | 4 | 0 | 10 | 50 | 30 | 28.51 |
| 20 | 6 | 10 | 20 | 37.5 | 90 | 86.89 |
| 21 | 8 | 0 | 30 | 25 | 150 | 43.25 |
| 22 | 6 | 10 | 20 | 37.5 | 90 | 83.84 |
| 23 | 6 | 10 | 20 | 37.5 | 150 | 93.17 |
| 24 | 8 | 10 | 20 | 37.5 | 90 | 79.70 |
| 25 | 6 | 10 | 20 | 37.5 | 90 | 81.49 |
| 26 | 4 | 20 | 30 | 50 | 30 | 81.30 |
| 27 | 4 | 10 | 20 | 37.5 | 90 | 85.10 |
| 28 | 6 | 10 | 20 | 37.5 | 90 | 84.79 |
| 29 | 8 | 0 | 10 | 25 | 30 | 25.60 |
| 30 | 8 | 20 | 30 | 25 | 30 | 75.40 |
| 31 | 6 | 10 | 20 | 37.5 | 90 | 85.55 |
| 32 | 6 | 10 | 20 | 37.5 | 30 | 96.45 |
| 33 | 6 | 10 | 20 | 37.5 | 90 | 84.43 |
| 34 | 6 | 10 | 30 | 37.5 | 90 | 90.55 |
| 35 | 6 | 10 | 20 | 37.5 | 90 | 84.10 |
| 36 | 8 | 0 | 30 | 50 | 30 | 24.37 |

| **Table S6.**  The ANOVA table of a face-centered central composite design (FCCD) for optimization of extraction step in an SBSE technique according to the obtained peak areas of 17-OHP. | | | | | | | |
| --- | --- | --- | --- | --- | --- | --- | --- |
| **Factors** | **Sum of square** | **Degree of freedom** | **Mean of square** | ***p***  **value** | **F value** | **Levels of factors** | |
|  |  |  |  |  |  | **Low** | **High** |
| **pH: A** | 7.66 | 1 | 7.66 | **0.0397** | 1.12 | 6 | 8 |
| **NaCl (%w/v): B** | 29246.90 | 1 | 29246.90 | **0.0000** | 4292.56 | 0 | 20 |
| **Sample volume (mL): C** | 83.26 | 1 | 83.26 | **0.0044** | 12.22 | 10 | 30 |
| **Extraction Temperature (°C): D** | 245.61 | 1 | 245.61 | **0.0001** | 36.05 | 25 | 50 |
| **Extraction Time (min): E** | 14655.90 | 1 | 14655.90 | **0.0000** | 2151.04 | 30 | 150 |
| **AA** | 32.37 | 1 | 32.37 | **0.0499** | 4.75 |  | |
| **AB** | 84.92 | 1 | 84.92 | **0.0041** | 12.46 |  | |
| **AC** | 103.33 | 1 | 103.33 | **0.0021** | 15.17 |  | |
| **AD** | 46.77 | 1 | 46.77 | **0.0224** | 6.85 |  | |
| **AE** | 20.73 | 1 | 20.73 | 0.1066 | 3.04 |  | |
| **BB** | 67.29 | 1 | 67.29 | **0.0085** | 9.88 |  | |
| **BC** | 13.08 | 1 | 13.08 | 0.1911 | 1.92 |  | |
| **BD** | 352.66 | 1 | 352.66 | **0.0000** | 51.76 |  | |
| **BE** | 5784.55 | 1 | 5784.55 | **0.0000** | 849.00 |  | |
| **CC** | 45.86 | 1 | 45.86 | **0.0235** | 6.73 |  | |
| **CD** | 12.94 | 1 | 12.94 | 0.1933 | 1.90 |  | |
| **CE** | 309.15 | 1 | 309.15 | **0.0000** | 45.37 |  | |
| **DD** | 151.18 | 1 | 151.18 | **0.0005** | 22.19 |  | |
| **DE** | 43.22 | 1 | 43.22 | **0.0270** | 6.34 |  | |
| **EE** | 731.96 | 1 | 731.96 | **0.0000** | 107.43 |  | |
| **Total** | 52335.70 | 32 |  |  |  |  | |
| **R^2^** | 99.84 |  |  |  |  |  | |
| **R^2^_adj_** | 99.58 |  |  |  |  |  | |

Based on the obtained results, equation 1 was proposed. In this equation, A was the pH value of the sample, B was the %NaCl (w/v), C is the volume of the sample solution (mL), D is the extraction temperature (°C), and E is the extraction time (min).

$\text{peak area =124.723 +145741 A - 1.8972 B - 2.04978 C - 8.88486 D +1.48649 E - 1.36208 }\text{A}^{\text{2}}\text{ + 0.133938 AB + 0.145815 AC - 0.0833852 AD + 0.0126117 AE +0.0437504 }\text{B}^{2}\text{ + 0.00529081 BC + 0.0405587 BD + 0.0310649 BE + 0.301725}\text{C}^{2}\text{ + 0.0101938 CD }\text{- 0.00795099 CE + 0.117235 D}^{\text{2}\text{ }}\text{+ 0.00269134 DE - 0.00731898 }\text{E}^{2}$ (1)

|  |
| --- |
| **Figure S1.** Pareto chart related to the optimization of the extraction step by central composite design. |

| 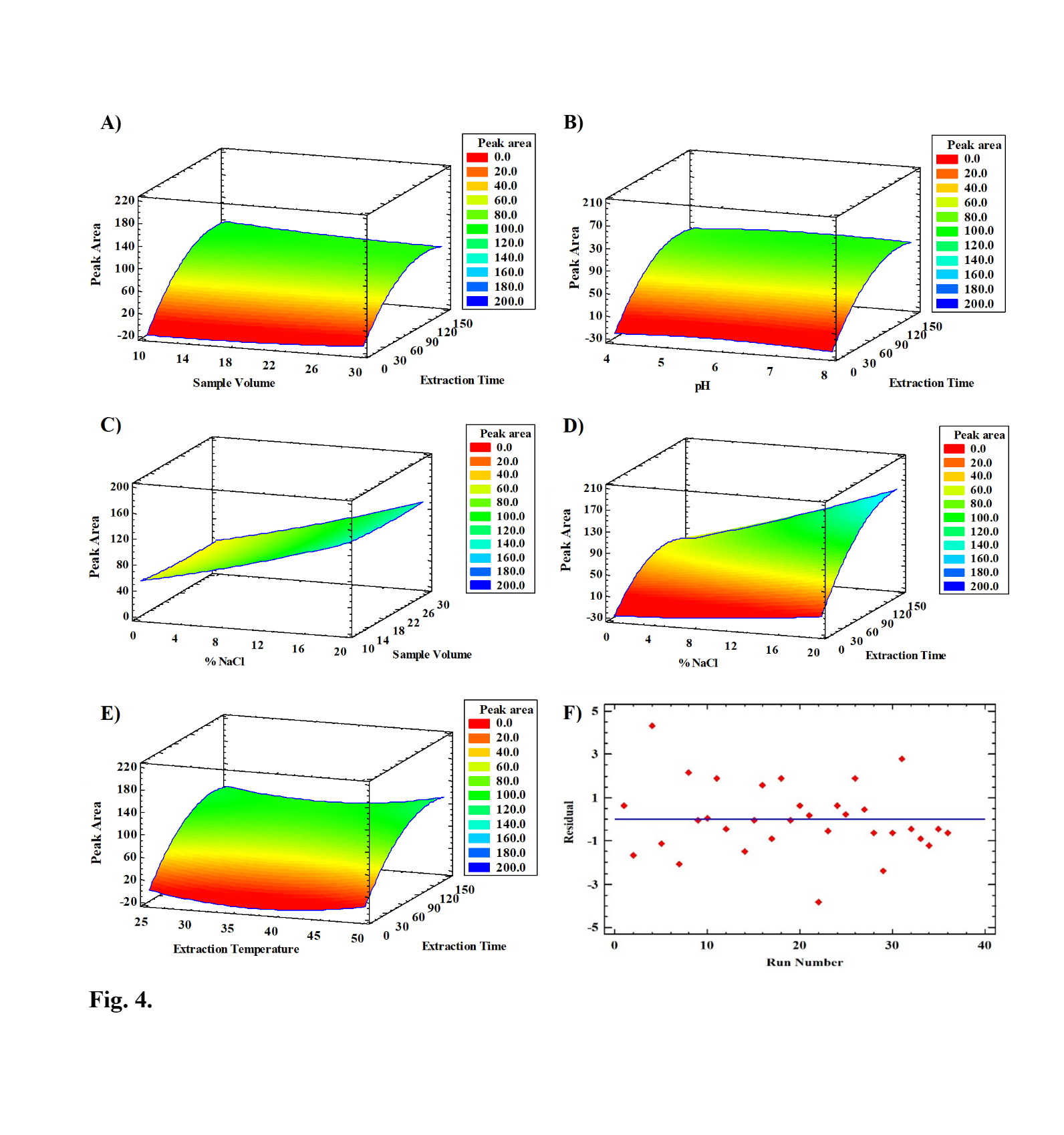 |
| --- |
| **Figure S2.** Plots related to the optimization of the extraction step by central composite design. The response surface plot for sample volume and extraction time (A), for pH and extraction time (B), NaCl (w/v%) and sample volume (C), NaCl (w/v%) and extraction time (D), extraction temperature and extraction time (E), and relative residual plot according to experiment number (F). |

| **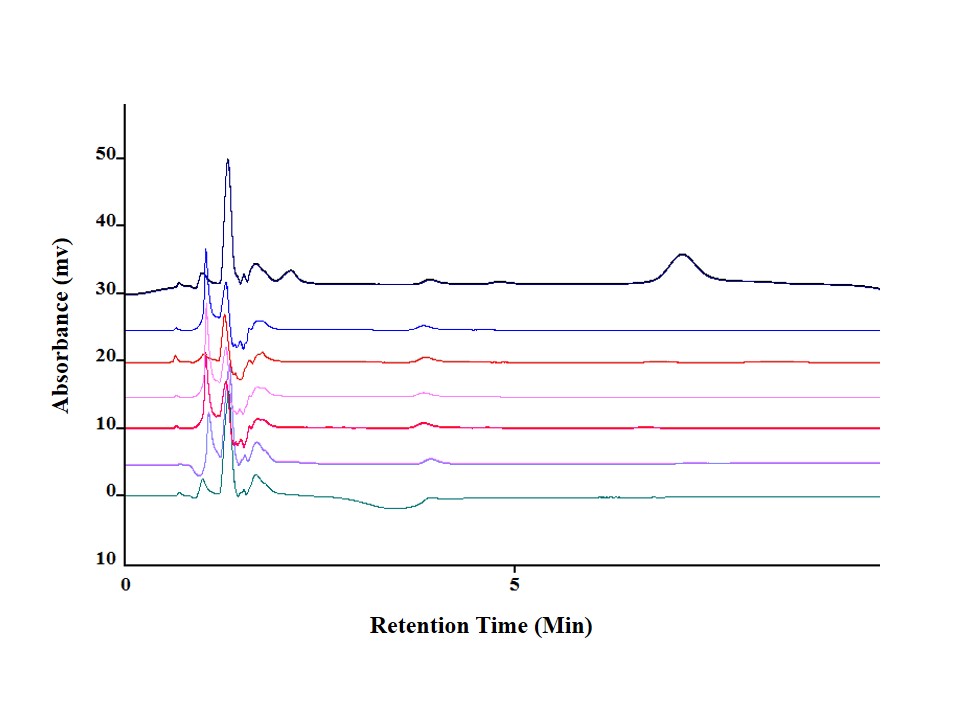**  **Time (min)** |
| --- |
| **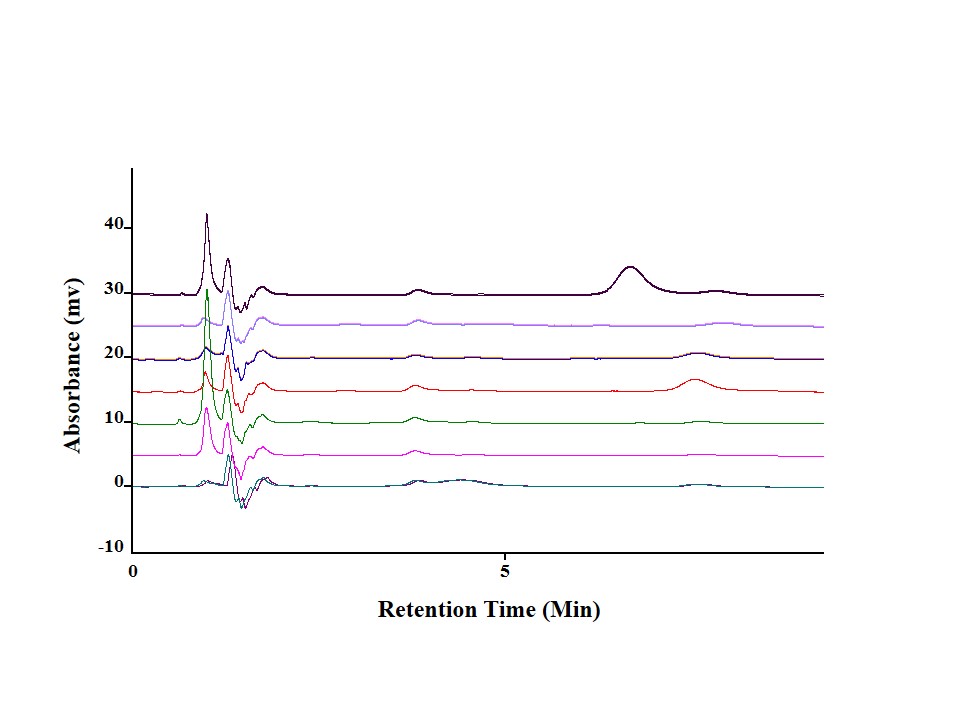**  **Time (min)** |
|  |

**Figure S3.** Chromatograms related to the analysis of 6 different real samples of water (A) and urine (B) to investigate the selectivity of the PDMS-SBSE-HPLC-UV method. The highest chromatogram of water and urine samples shown spike 500 ng/mL 17-OHP to a blank sample of water and urine respectively.

|  |
| --- |
| **Fig. S4.** Reusability of PDMS stir bar in extraction of 17-OHP from water during 18 independent extractions under constant extraction conditions. UCL & LCL shown upper control limit and lower control limit, respectively. |

| **Table S7.** ANOVA table of calibration model of 17-OHP in water and urine samples. | | | | | |
| --- | --- | --- | --- | --- | --- |
| **samples** |  | **df** | **SS** | **MS** | **F** |
| **Water** | **Regression** | 1 | 170812 | 170812 | **105356.35** |
|  | **Residual** | 19 | 31 | 2 |  |
|  | **LOF** | 5 | 18 | 4 | 4.200 |
|  | **PE** | 14 | 12 | 1 |  |
|  | **Total** | 20 | 170843 |  |  |
| **Urine** | **Regression** | 1 | 82049.6 | 82049.6 | **51.4514** |
|  | **Residual** | 22 | 350.8 | 15.9 |  |
|  | **LOF** | 6 | 207.8 | 34.6 | 3.870 |
|  | **PE** | 16 | 143.0 | 8.9 |  |
|  | **Total** | 23 | 82400.5 |  |  |
| **F (5,14) at 99% confidence level = 4.695**  **F (6,16) at 99% confidence level = 4.202** | | | | | |

| **Table S8.** Recovery and RSD percentage values for extraction and analysis of real samples using PDMS-SBSE-HPLC-UV method | | | | |
| --- | --- | --- | --- | --- |
| **Real Sample** | **Added amount of 17-OHP (ng/mL)** | **The calculated concentration (ng/mL)** | **Recovery%** | **RSD%^a^** |
| Tap water | 0 | ND^b^ | - | - |
|  | 500 | 494 | 98 ± 1 | 1 |
| Well water | 0 | ND | - | - |
|  | 500 | 502 | 100 ± 1˃ | 1˃ |
| Urine | 0 | ND | - | - |
|  | 500 | 529 | 105 ± 1˃ | 1˃ |
| ^a^ Relative standard deviation  ^b^ Not detected | | | | |
